# Deciphering Molecular Mechanisms of Interface Buildup and Stability in Porous Si/Eumelanin Hybrids

**DOI:** 10.3390/ijms18071567

**Published:** 2017-07-19

**Authors:** Elisa Pinna, Claudio Melis, Aleandro Antidormi, Roberto Cardia, Elisa Sechi, Giancarlo Cappellini, Marco d’Ischia, Luciano Colombo, Guido Mula

**Affiliations:** 1 Dipartimento di Fisica, Università degli Studi di Cagliari, S.P. 8 km 0.700, 09042 Monserrato, Italy; Aleandro.antidormi@dsf.unica.it (A.A.); roberto.cardia@dsf.unica.it (R.C.); elisa.sechi85@gmail.com (E.S.); giancarlo.cappelini@dsf.unica.it (G.C.); luciano.colombo@dsf.unica.it (L.C.); guido.mula@unica.it (G.M.); 2 Istituto Officina dei Materiali CNR-IOM, Unità di Cagliari SLACS, Cittadella Universitaria di Monserrato, S.P. 8 km 0.700, 09042 Monserrato, Italy; 3 Department of Organic Chemistry and Biochemistry, University of Naples “Federico II”, Via Cintia 4, 80126 Naples, Italy; dischia@unina.it

**Keywords:** porous silicon, organic/inorganic hybrids, eumelanin, density functional theory, optical properties of materials

## Abstract

Porous Si/eumelanin hybrids are a novel class of organic–inorganic hybrid materials that hold considerable promise for photovoltaic applications. Current progress toward device setup is, however, hindered by photocurrent stability issues, which require a detailed understanding of the mechanisms underlying the buildup and consolidation of the eumelanin–silicon interface. Herein we report an integrated experimental and computational study aimed at probing interface stability via surface modification and eumelanin manipulation, and at modeling the organic–inorganic interface via formation of a 5,6-dihydroxyindole (DHI) tetramer and its adhesion to silicon. The results indicated that mild silicon oxidation increases photocurrent stability via enhancement of the DHI–surface interaction, and that higher oxidation states in DHI oligomers create more favorable conditions for the efficient adhesion of growing eumelanin.

## 1. Introduction

Organic–inorganic hybrid devices attract growing research and industrial interest for a broad range of applications since they combine the low fabrication costs and versatility of the organics with the usually higher efficiency of the inorganics [1,2,3]. A special position in this context is occupied by eumelanin-based hybrid materials, which offer unexplored bioinspired and biocompatible opportunities for technologically advanced devices. Eumelanins are a black to dark brown insoluble variant of melanin pigment that arise by the oxidative polymerization of tyrosine via 5,6-dihydroxyindole intermediates, and are responsible for dark coloration in human and mammalian skin, hair, and eyes. Because of the amorphous character and the complete insolubility in all solvents, eumelanin structure is still under assessment despite important advances in recent years [4,5,6]. Also, the biological role of eumelanins is still a matter for debate, although there is general agreement that these pigments mainly serve a photoprotective function and contribute to the antioxidant defense against oxidative stress. Most eumelanin biological functions are due to their peculiar physicochemical properties, which include broadband absorption in the visible region [7,8,9,10], a stable free radical character [1,2,3,11], and hybrid water-dependent ionic electronic conductor behavior [12,13]. Available evidence indicates that eumelanin broadband absorption, free radical, and electrical properties can be accounted for by a complex interplay of aggregation-dependent intermolecular perturbations of π–electron systems between the main oligomer components, subject to structural and redox control [14,15,16,17,18]. However, several crucial gaps still hinder a detailed understanding of the structural, electronic, and optical properties of eumelanins. Settling open issues relating to the mechanisms of buildup and control of interface interactions is therefore a central priority for the design of innovative bioinspired hybrid systems for optoelectronic and organic bioelectronics applications.

Recently, we reported a promising organic–inorganic hybrid material for photovoltaic applications consisting of a bulk heterojunction of porous silicon (PSi) and eumelanin obtained by polymerization of the key precursor 5,6-dihydroxyindole (DHI) [19]. DHI was introduced into the columnar PSi pores and then polymerized to form eumelanin [11]. The relatively intense absorption of eumelanin in the near-infrared region was found to enhance the light absorption capabilities of the empty porous silicon matrix, increasing significantly the photocarrier collection efficiency at longer wavelengths [11].

In a subsequent variant of that methodology, the polymerization of DHI in *n*^+^-doped porous silicon (*n*-PSi) was produced in situ by ammonia-induced solid state polymerization (AISSP) [20] after the pores were filled with DHI [12]. With this hybrid, a relatively intense photocurrent density up to 3.8 mA·cm^−2^ was measured upon irradiation with visible light. The photocurrent was not affected by acetic acid vapors but was irreversibly abated by gaseous ammonia, supporting the potential of eumelanin as a powerful enhancer of PSi photoresponse to visible light via hole-type electrical conduction. However, despite the promising features of the PSi–eumelanin interface produced by the AISSP protocol, several issues remained to be settled including the low efficiency, a marked instability, and a limited reproducibility.

To unravel the complexity of the hybrid eumelanin/Si interface to design efficient and stable PSi–eumelanin-based devices, it is therefore essential to elucidate the molecular mechanisms of eumelanin buildup and adhesion on the inorganic substrate. One main goal is to improve the adhesion, interaction and electron-ion transfer at the organic/inorganic interface in order to optimize the electronic performance for photovoltaic applications.

The aim of this paper is to address the factors responsible for the main interface issues, including temporal instability [11,12] of the bulk heterojunction between PSi and eumelanin. Reported herein are: (a) a rational strategy to modify the silicon surface to enhance adhesion and increase stability; (b) the preparation of a chemically manipulated eumelanin via inclusion of carboxylated indole units to enhances the anchoring with the silicon surface; (c) a spectrophotometric investigation of DHI oxidation and eumelanin formation in ethanol as an organic solvent, to gain an insight into the nature of the chromophoric species that develop from DHI polymerization in a relatively hydrophobic environment like the silicon surface; (d) a computational analysis of a representative oligomer component of eumelanin at various oxidation levels, to compare its predicted absorption properties with those of the oxidation reaction in ethanol and to assess the efficiency of interaction and adhesion with silicon as a function of the oxidation state. The “in silico” modeling approach reported herein was based on a solid background of experimentally determined structures and provides a useful entry to address hybrid interface issues [21,22].

## 2. Results and Discussion

### 2.1. Temporal Stability of the Eumelanin-PSi Hybrids

**Figure 1:**
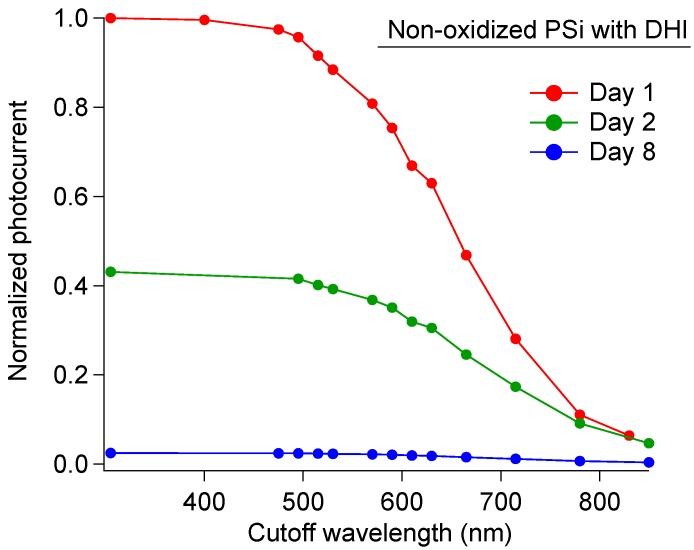
Normalized photocurrent measurements obtained in three different days on the same PSi/eumelanin sample.

One of the main issues with eumelanin–PSi hybrids is their short lifetime (a few days) was photocurrent generators and a significant fluctuation of the photocurrent values for different samples (about +/− a factor of three). Although the high reactivity of the developed surface of pristine PSi samples is a well-known characteristic of this material [23,24], this ageing effect depends strongly on the surface treatment. Several techniques have been used to stabilize the PSi surface, from oxidation [25] to hydrocarbonization [26] to organic grafting [27,28]. In our case, the freshly prepared PSi samples were rapidly (within one hour) impregnated with DHI/EtOH solution, and the polymerization process followed immediately after the impregnation. This rapid impregnation strongly limits the exchanges with the atmosphere both by the pore-filling itself and by the formation of bonds between DHI and PSi. In Figure 1 we show the typical temporal evolution of the photocurrent measured at day 1 (the maximum of this curve has been used as a normalization factor) and days 2 and 8 of a eumelanin–PSi hybrid prepared as described in [11,12]. The photocurrent is measured using low-pass filters following the methodology also described in [11,12]. With this method, an increase of the photocurrent value when changing filters from longer to shorter cutoff wavelengths is expected, given the increase in the impinging light wavelength range. The curves in Figure 1 show light absorption and photocurrent generation in a large part of the wavelength range we studied (outside this range the light source intensity was insufficient to give rise to significant photocurrents). The photocurrent value at the shortest wavelength represents the “white light” photocurrent. The short lifetime of the system is evidenced by the almost 60% decrease of the photocurrent observed at day 2 with respect to day 1, and the almost zero photocurrent measured at day 8. The dark current value has been measured for each sample and was about the same, within the bounds of experimental error, as the photocurrent corresponding to the 830 nm measurement. A direct comparison of the absolute photocurrent results is presently not useful since, as already reported, these junctions show a significant fluctuation in the photocurrent from sample to sample with respect to other polymers [29] and a comparison of the relative temporal variation of the photocurrent with respect to the initial value is much more effective as a comparison method.

Several factors can contribute to this behavior, including the formation of the organic/inorganic interface in a porous structure, the polymerization process of eumelanin from DHI, and the loss of hydration water. To address the complexity of hybrid formation in a porous structure, this issue was addressed by investigating the PSi/eumelanin interface and analyzing the polymerization process from DHI to eumelanin.

### 2.2. Effect of Surface Modifications on the Temporal Stability of the Photocurrents

Interface instability can be related to the lack of formation of stable bonds between Si and eumelanin. Based on this assumption, one possible explanation is that the volume reduction accompanying polymerization to eumelanin would weaken the initial electrical contact between PSi and the organic eumelanin component, lowering photocurrent generation.

To test this hypothesis, Electrochemical Impedance Spectroscopy (EIS) was used to gain information on the interface stability. EIS is very sensitive to surface and interface processes, and this makes it very useful for porous materials because of their high developed inner surface [30].

**Figure 2:**
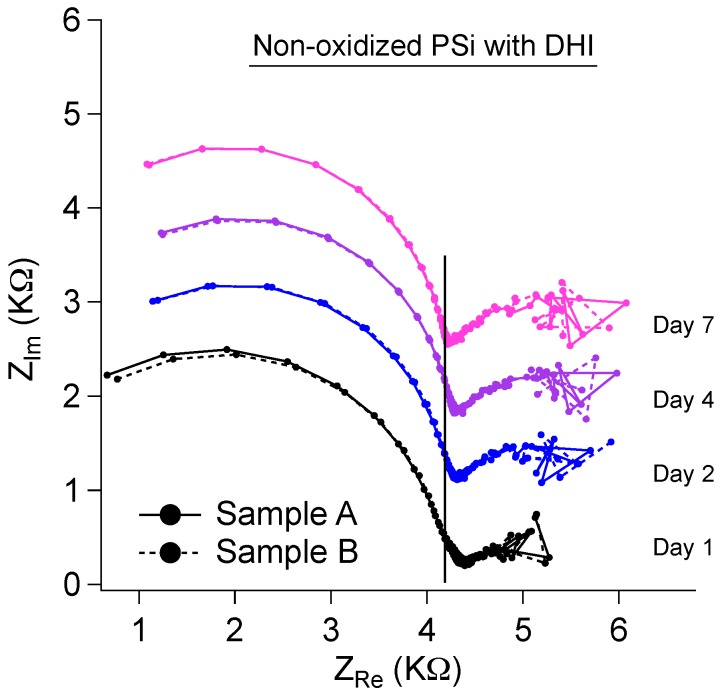
GEIS (Galvanostatic EIS) measurements of two standard PSi/eumelanin samples over a week.

In the hypothesis of a significant role played by the PSi/melanin interface, a different behavior should be obtained when modifying the interface formation process. As a reference measurement before any surface modification, we studied the behavior of standard PSi/melanin samples. In Figure 2 we show the Galvanostatic EIS (GEIS) curves of two samples. The GEIS data are represented in a Nyquist plot, which is a plot of the imaginary part of the impedance vs. the real part. The frequency, not visible in this kind of plot, goes from the highest frequencies (100 kHz) on the left side to the lowest (100 mHz) to the right. The curves for each day following the first have been vertically translated for better readability. All curves have also been aligned to the right end of the high-frequency semicircle (the left one) of the first measurement since the differences in its position are related only to the series resistances that depend on the contact and not on the samples’ characteristics. The fluctuation in the real measurements was in any case within 200 Ω. The same procedure has been adopted for all the GEIS graphs shown in this work. Although quantitative information about interface modification can be gained by fitting the GEIS curves, this will be the focus of future studies. Here, we aim at assessing whether there are differences in the overall impedance response from samples that underwent different processes. As described later, the main difference among the samples is the presence or absence of an additional resonance and the stability with time of the impedance response. This information, although qualitative, gives a clear indication of the stability differences induced by the different processes of interface formation, and is sufficient to select the best surface treatment procedure among those examined in this study. 

The first information that can be obtained from Figure 2 is the very high reproducibility in the samples’ behavior, given the almost perfect superposition of the curves related to the two samples. This is a key parameter when comparing results for different samples and indicates that the variations in the photocurrent behavior of the samples are probably also related to the polymerization process.

In the day 1 curves, in black in the figure, we can see a large semicircle on the left, corresponding to higher frequencies, a small part of a middle semicircle (on the right of the vertical line) and, on the right, another semicircle, smaller than the first one, for lower frequencies. In the measurements made on days 2, 4, and 7, we can see the disappearance of the middle circle (at about 4300 Ohm) and the rapid growth of the last one. We can interpret this rapid evolution of the circles as a sign of the instability of the porous silicon/DHI interface, which significantly changes in the first 4–7 days from preparation and confirms the instability in the photocurrent measurements.

To probe the interface formation process, we used two approaches. The first was based on a slight electrochemical oxidation of the silicon surface, the second used a 50/50 mixture of DHI and 5,6-dihydroxyindole-2-carboxylic acid (DHICA) instead of pure DHI to impregnate the sample.

#### 2.2.1. Effect of PSi Surface Oxidation

**Figure 3:**
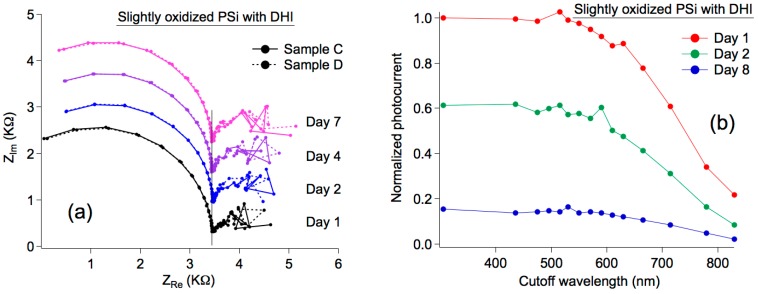
Behavior of PSi/eumelanin samples with a slight surface oxidation (3%, as defined in the text) of PSi before impregnation. (**a**) GEIS measurements; (**b**) normalized photocurrent measurements.

Partial oxidation of the surface was expected both to exert a stabilizing effect and to facilitate a stronger bonding of DHI to the surface, thanks to better affinity between SiO_2_ and DHI. The potential advantages of a thin oxide layer in hybrid and Si/organic photovoltaic heterojunction have already been described in several publications [31,32,33,34,35]. In those works, it appears that there is an optimal thickness (about 1 nm) for the silicon oxide interfacial layer, with the main limit being that an excessive thickness would limit the conduction and worsen the photovoltaic performance. In this case, the main purpose was to change the surface chemistry only slightly. For this reason, we limited our electrochemical oxidation (EO) to 3% of the maximum achievable with our experimental setup. Since we use a constant current EO [36], the maximum oxidation is achieved for a process duration equal to the time needed to reach the maximum instrumental voltage (10 V). The 3% oxidation level is then obtained by an EO duration equal to 3% of this time. The maximum oxygen content for the EO in our experimental setup, measured by Scanning Electron Microscopy—Energy Dispersive Spectroscopy (SEM-EDS) was equal to 50% of the total silicon and oxygen atoms. A 3% oxidation will then lead to a percentage of oxidized Si atoms of less than 2%. A direct measurement of the oxide layer thickness within the porous matrix is an extremely complex task, given the pores’ structure and size, and seems therefore beyond the scope of this work. Figure 3a shows the GEIS curves of two mildly oxidized (3%) standard PSi samples, impregnated with DHI. As previously noticed, a high reproducibility of the samples was observed. With respect to non-oxidized PSi/DHI samples, the middle semicircle was apparently absent and the semicircle at lower frequencies displayed slower evolution. Thus, mild oxidation of the surface leads to a much slower evolution of the GEIS behavior over time with respect to the reference sample.

Figure 3b shows the photocurrent decay of a slightly oxidized sample over a week. The data show an improvement in the stability of the photocurrent values compared with standard samples, as judged by the 40% decay over the first two days versus a 60% decay without treatment. Moreover, after one week the intensity of the photocurrent was higher than 15% of the initial value. To fully appreciate this effect, it should be considered that a typical non-oxidized sample shows photocurrent intensities as low as about 1% or less of the initial value after a few days. This improved stability is also reproducible, indicating real improvement rather than a random result.

**Figure 4:**
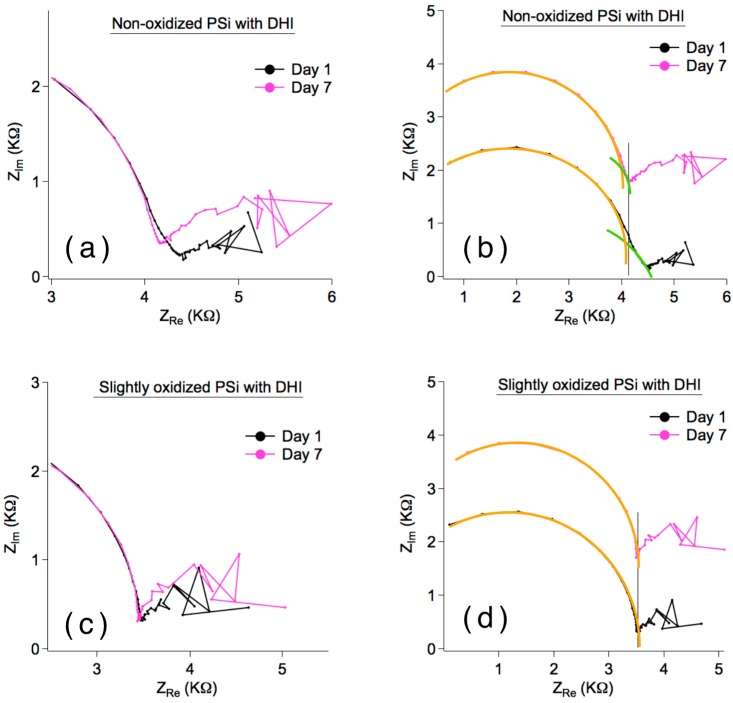
Graphical analysis of the GEIS results of Figure 2 (panels (**a**,**b**) and of those of Figure 3a (panels (**c**,**d**)). The results of the first and seventh days are shown. In panels (**a**,**c**) are reported the enlarged views of the data of Figure 2 and Figure 3a, respectively. In panels (**b**,**d**) partial ellipses are used as a visual guide to show the presence of an additional resonance in the non-oxidized samples (**b**) with respect to the slightly oxidized samples (**d**).

A few more comments are needed for the comparison of the results in Figure 2 and Figure 3a. In Figure 4 we report an enlarged view of the first and seventh days GEIS results for (Figure 4a) a non-oxidized sample (from Figure 2) and (Figure 4c) a slightly oxidized sample (from Figure 3a) to evidence the behavioral differences between these two kinds of samples. Given the high reproducibility of the results of Figure 2 and Figure 3a, only the results of a single sample have been reported in each panel for a clearer comparison. In Figure 4b,c we superimposed partial ellipses on the experimental results as a visual guide. It is evident that while two ellipses are needed to obtain a good superposition to the experimental data for non-oxidized samples (Figure 4b), a single curve is needed for the slightly oxidized samples (Figure 4d). This is a clear indication that without the oxidation an additional resonance is present in the samples’ frequency response, even if not fully separated from the higher frequency resonance. The effect of the oxidation is to suppress this additional resonance and give the samples a constant impedance response over the periods of time explored in this study.

#### 2.2.2. Effect of the Use of a DHI and DHICA Mixture for the Impregnation

The second strategy tested as a possible means of stabilizing the junction was based on the use of mixed solutions of DHI and DHICA to impregnate the PSi matrices. The rationale was that DHICA combines a DHI skeleton with a carboxyl function that could improve the anchoring on the PSi surface via oxygen bridges akin to those observed for Ti oxide surfaces [37]. Moreover, the same carboxyl function may be responsible for a decreased oxidizability of DHICA with respect to DHI, preventing the catechol function to be oxidized to the quinone form on the Si surface [38]. The overall effect of the DHICA within the pores would thus be to strengthen the eumelanin–PSi junction, increasing interface stability and the charge transport after the exciton separation.

**Figure 5:**
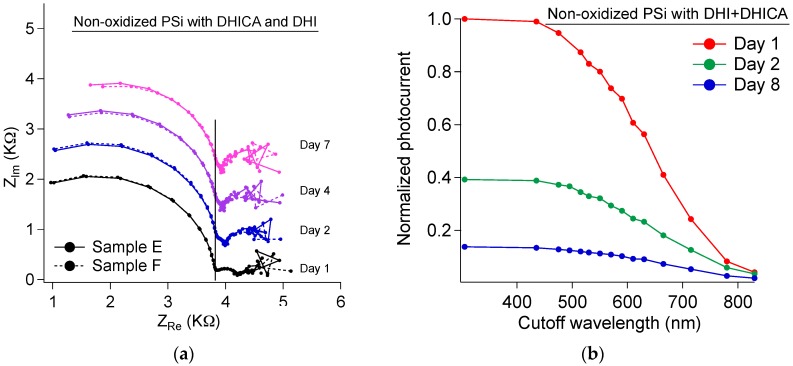
Behavior of PSi/eumelanin samples prepared using a 50/50 DHI/DHICA mixture. (**a**) GEIS measurements; (**b**) normalized photocurrent measurements.

Figure 5 shows the behavior of two samples prepared using standard PSi and the DHI/DHICA mixture. A high reproducibility of the fabrication process was noticed, as demonstrated by the superposition of the GEIS curves shown in Figure 5a. Although in principle DHICA should improve the stability of the junction, experimentally we were not able to demonstrate a significant improvement with respect to the PSi/DHI case. In fact, there is still a decrease in the photocurrent values by about 60% in only two days (Figure 5b), even if after a week the reduction is more similar to the slightly oxidized samples, and the evolution of the GEIS semicircles over a time span of seven days is even larger than that shown in Figure 2.

On the basis of these results it was concluded that a modest stabilization of the PSi/melanin interface can be achieved by mild oxidation of the PSi layer before impregnation with the monomer, and that the addition of DHICA slightly modifies the GEIS semicircle profile but does not increase the stability.

### 2.3. Oxidation of DHI in Ethanol Solution

**Figure 6:**
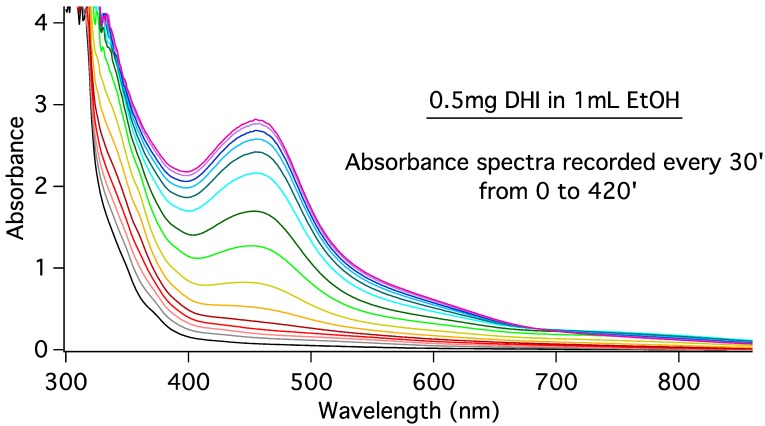
Absorbance spectra of ethanolic DHI solutions with a concentration of 0.5 mg/mL.

To gain an insight into the molecular mechanisms leading to interface formation between growing eumelanin on the PSi surface, in model experiments the evolution of the absorbance spectra in air-equilibrated solutions of DHI in ethanol at various concentrations was examined. In Figure 6 we report the results for a concentration of 0.5 mg of DHI in EtOH. Selection of these reaction conditions was suggested by the opportunity to consider the relatively hydrophobic nature of the PSi substrate on which interface formation occurs, which could not be aptly mimicked by the usual aqueous solution experiments. Moreover, a brief insight into the oxidation behavior of DHI in an organic solvent was encouraged by the apparently scanty and scattered literature on this topic.

From the data in Figure 6, absorbance variations were determined at three different wavelengths corresponding to a shoulder in the 320–380 nm region, the main band of the spectra (460 nm), and a weaker band centered at 780 nm. This behavior is not modified for different concentrations (Figure S1).

**Figure 7:**
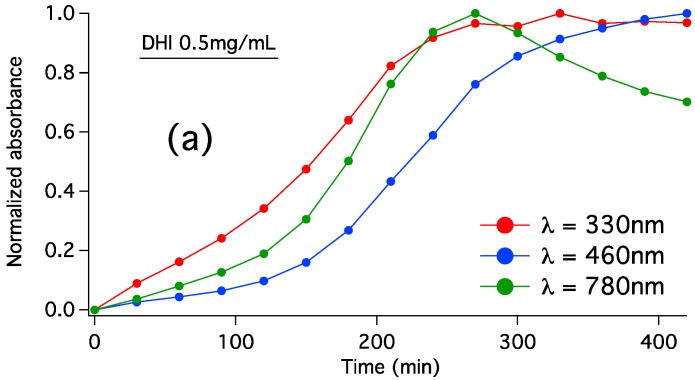

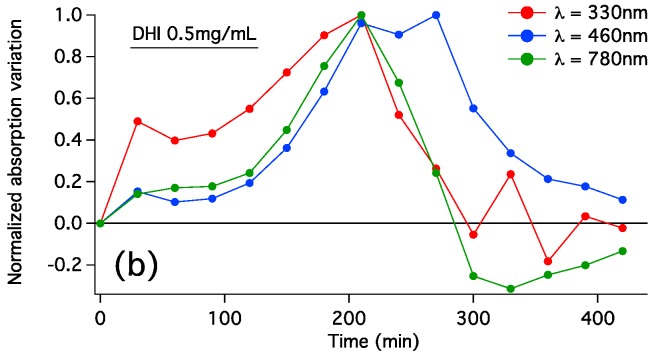
Analysis of the evolution of the absorbance spectra of Figure 5 at three different spectral positions. (**a**) Normalized evolution of the absorbance; (**b**) absorbance increase in each time step, normalized with respect to the maximum positive value for each curve.

In Figure 7 we report the variation of the absorbance at these three wavelengths obtained from the data used for Figure 6. In Figure 7a we report the absorbance variation with respect to its initial value at the given wavelength, normalized with respect to the maximum increase for each curve for an easier comparison. In Figure 7b we report the absorbance increase in each step, normalized with respect to the maximum positive value for each curve. The data show that the band at 330 nm increases at a faster rate, followed by the bands at 780 and 460 nm peaks, in that order. Notably, a rapid decrease was apparent in the absorption at 780 nm, while other bands are still growing. Absorbance variations recorded at each measurement show, moreover, that bands at 330 and 780 nm attain their maximum at the same time despite a delayed increase of the latter, and that the band at 460 nm is the last to reach its maximum value. A similar behavior was observed for all DHI concentrations studied (see Figure S2). It can thus be concluded that the three bands correspond to different yet related species that are likely to correspond to dimers/oligomers of DHI in oxidized forms. To decipher the complex sequence of event underlying the observed spectral changes, it may be useful to refer to a recent investigation of DHI oxidation in aqueous buffer [16]. Under such conditions, DHI oxidation was found to give rise to a broad visible chromophore around 560 nm, which was attributed to mixtures of dimers and higher oligomers, and the analysis was not pushed further under those conditions. 

From the experiments reported herein, it can be concluded that DHI also polymerizes in an air-equilibrated organic medium, and that the process leads to the formation and evolution of more defined chromophoric species compared to the same reaction in aqueous neutral buffer, with less pronounced scattering at longer wavelengths. Such differences can be explained by considering that in ethanol oligomer aggregation and precipitation are not so pronounced as in an aqueous buffer, where significant scattering is observed. Moreover, the lower dielectric constant in an organic solvent would not favor oligomer ionization by deprotonation as in an aqueous buffer. On this basis, the higher absorption wavelength and flatter appearance of the main visible band in the aqueous buffer could be ascribed to the deprotonated forms of the oxidized oligomers. This conclusion was supported in a separate experiment in which the oxidation mixture of DHI in aqueous buffer pH 7.0 was acidified to pH 2, causing a marked blue shift of the broad absorption band at 560 nm to about 500 nm. It is possible that the transient species absorbing at 780 nm is an unstable precursor of the main band at 460 nm, but no evidence supports this conclusion. The longer persistence of the main chromophores in ethanol with respect to the aqueous buffer can be explained by the stronger solvation effects in the organic solvent, limiting aggregation and precipitation of dark material.

Based on this background of experimental and model studies, further insights into the process of interface buildup were gained by computational modeling of the formation and adhesion of single and doubly stacked molecules reported in this work, allowing for a direct comparison of the stacking and covering processes of eumelanin on Si. To this aim, the chromophoric changes observed in ethanol were used as a basis to validate the model structure selected to probe eumelanin adhesion on Si by computational methods.

### 2.4. Formation and Stacking Energies

The structural variety of the isomers that populate each oligomer level contributes to the molecular disorder of eumelanin type materials [4,9,19,39]. In addition, eumelanin structures can differ also in their redox state, which, for each DHI monomer unit, comprises the catechol form as well as the 2-electron oxidation state (5,6-indolequinone, IQ) and its tautomers, the quinonemethide (QM) and the quinoneimine (QI). The variety of structures accessible for the various redox levels (semiquinones are not considered for the sake of simplicity) contribute to the second level of disorder in eumelanins, i.e., electronic disorder. Both molecular and electronic disorder are considered to determine the overall properties of eumelanin, whereby control of disorder is a promising approach for tailoring eumelanin properties [19].

**Scheme 1:**
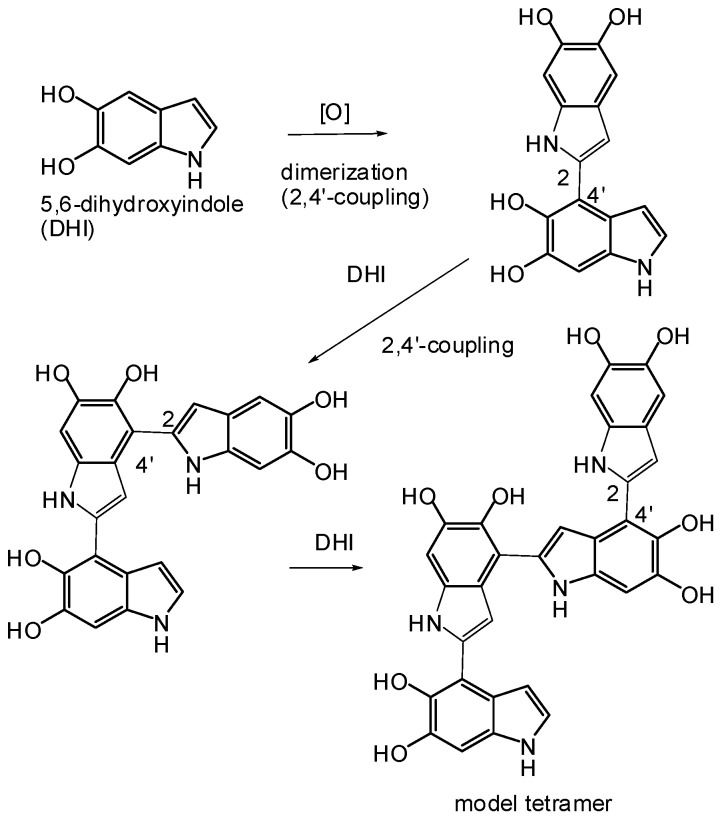
Mechanism of oxidative coupling of DHI leading to isolated 2,4′-dimer and 2,4′:2′,4″ trimer and model tetramer (Model 1).

For the purposes of this study, 2,4′:2′,4′′:2′′,4′′′-tetraindolyl (Model 1) was considered as a model representative component of eumelanin for computational analysis (Scheme 1). Selection of a tetramer as a model eumelanin building block was the result of a compromise between the need for a sufficient level of molecular complexity and the computational costs associated with higher oligomers. The model tetramer (Scheme 1) was built upon an extension of the coupling sequence leading to the 2,4′-dimer, the major intermediate isolated from the oxidation of DHI, and the related trimer 2,4′:2′,4′′-terindolyl, isolated in early studies. It was considered a suitable representative of the various possible tetramers because of the lack of specific symmetry properties and its putative origin from major isolated DHI-based oligomers. It is worth mentioning that the direct oxidation of dimer 2,4′ leads to a tetramer with an anomalous 2,3′ bonding (2,4′:2′,3′′:2′′,4′′′-tetraindolyl), which, however, was not considered because of the specific reaction conditions used in that study [19].

A more complete analysis of disorder in eumelanins would encompass the enormous number of possible molecular models and alternative redox states, and was outside the scope of the present investigation because of the huge computational effort required. 

**Figure 8:**
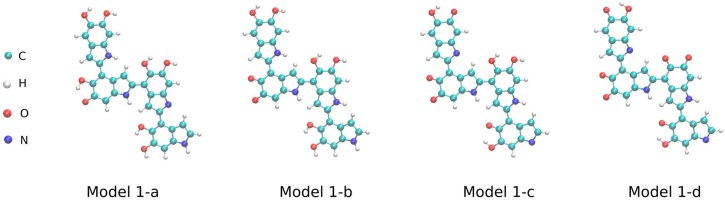
Different oxidation states of the model tetramer selected for computational studies. Model 1-a (2-electron oxidation, 1 C=O group); Model 1-b (2-electron oxidation, 2 C=O groups), Model 1-c (six-electron oxidation, 4 C=O groups); Model 1-d (eight-electron, 6 C=O groups).

All the results were obtained from Density–Functional Theory, employing the plane–wave Quantum Espresso package with the PBE (Perdew, Burke and Ernzerhof, [40,41]) exchange–correlation functional. For other computational details, refer to [42]. For Model 1 tetramer, four different oxidation states were considered, which are schematically depicted in Figure 8. They correspond to two tautomers of the putative two-electron oxidation product, and one tautomer each for the six-electron and eight-electron oxidation products.

The formation energies of these molecules are given in Table 1 in a vacuum, methanol, and water. The choice of methanol as a solvent was guided by an attempt to interpret spectral changes accompanying DHI oxidation in ethanol and the results are compared with those in water.

Formation energies are calculated as $E_{tetramer}+3E_{H_{2}}-\underset{monomer}{\sum }E_{monomer}$, e.g., as the difference between the molecule energy $E_{tetramer}$ and the sum of the monomeric energies $E_{monomer}$ (the energy of the molecular hydrogen $E_{H_{2}}$ released in the reaction is also included).

The results show a clear dependence of the formation energies on the solvent used in the synthetic process. The presence of solvent, here taken into account via the dielectric permittivity of the medium, increases the formation energy, causing a decrease in the molecular stability. Some of the structures considered are not formed in methanol (ε=32.7), while none is stable in water (ε=80.1). 

Moreover, the decrease in molecular stability in solvents with high permittivity is quite general and is also found for doubly stacked molecules.

Another relevant piece of information is that molecules with a high number of carbonyl (C=O) groups yield improved stability, as opposed to –OH groups, which determine a higher formation energy. However, in that case the result can be at least partially due to the fact that for steric reasons two nitrogen atoms at most can be present as N–H groups. Looking more carefully at the results of Table 1, it seems clear that what is really important in determining the stability is the number of single carbonyl (quinoneimine) units, while *o*–quinone units do not appear to contribute further to the overall stability.

For the molecular structures outlined above, stacking energies have also been computed. Values are given in Table 2. Defined as $E_{2tetramers}-2E_{singletetramer}$, stacking energy expresses the tendency of the considered molecules to form a stacked structure. Negative values are found for all the molecules considered, highlighting how the molecules naturally tend to bind to each other via π–π stacking.

### 2.5. Calculation of the Tetramer Optical Absorption 

To assess whether structures related to the model tetramer may be responsible for the spectrophotometric course of DHI oxidation in ethanol and hence for the buildup of the eumelanin–silicon interface, the absorption spectrum of the model tetramer in Figure 7 was calculated at the highest oxidation level examined, i.e., the eight-electron oxidation state (Model 1-d). Calculated spectra range from the Near Infrared (NIR) to the border of Mid/Far Ultraviolet (MUV/FUV). In addition, as a benchmark to validate the computational methodology, one of the most studied hypothetical porphyrin-type cyclic tetramers (PT) proposed by Kaxiras and coworkers [43,44] was also investigated.

In the study by Meng and Kaxiras in [44], a calculated absorption spectrum of the PT structure was presented, using a TD–DFT scheme in the PBE approximation for the exchange and correlation potential, as implemented in the SIESTA computational code [45]. In principle, our choice of B3LYP should be more suitable for describing the electronic and optical excitations in molecules [46]. A comparison of our spectrum and theirs is available in the Supplementary Materials (Figure S3).

**Figure 9:**
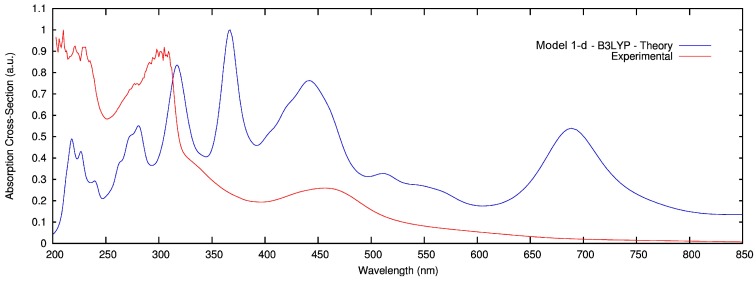
Comparison between the calculated spectra for the Model 1-d structure (blue line) and the experimental absorption of a 0.25mg/mL solution of DHI in EtOH (red line, curve corresponding to the absorbance after 600 min).

Figure 9 reports the calculated absorption spectrum for the tetrameric structure modeled in the present paper and the experimental absorbance measured for DHI in EtOH at 0.25 mg/mL concentration 600 min after preparation. The theoretical curve is normalized to have the same maximum height of the experimental output for easier comparison.

Under the specific experimental conditions described above, DHI monomers undergo an aggregation process that causes changes in the absorption spectrum of the solution as a function of time.

Within the gross limitations of the present approach, due to the marked complexity of DHI polymerization and the notorious difficulty of modeling the myriad structures and redox states available to each aggregate of DHI oligomers, it appears that calculated model structures show absorption bands spanning the entire visible range, in line with what is observed in a DHI polymer in ethanol. A superposition of the computed spectra is shown in the Supplementary Materials (Figure S4). 

As is clear from Table 3 for both tetrameric forms, the onset structure is made mainly by HOMO → LUMO electronic transitions and takes place in the near infrared region (NIR). The first peak in the wavelength range considered (labeled B in Table 3) lies for both tetramers at the border of the NIR/visible spectra and is composed of different transitions. The maximum peak (labeled A in Table 3) is positioned for both tetrameric structures at the high-energy edge of the visible/near-UV spectra. For PT, different transitions contribute to that peak, while for the Model 1-d tetramer it originates mainly from HOMO → LUMO + 4 electronic transition.

In Table 4 we reported the position in wavelength (nm) of the main structures present in the three curves reported here: the two calculated for the PT and Model 1-d tetramers and those observed in the experimental measurements.

The PT tetramer shows a strong peak A (see the Supplementary Materials for the absorption spectrum) very near to an experimental local minimum (400 nm), while the PT theoretical local maximum B at 800 nm does not seem to have an experimental counterpart. On the other hand, in the case of Model 1-d the main structures A and B, aside from differences in oscillator strengths, show moderate matching with the experimental shoulders found at 340 and at 700 nm, respectively.

After Table 4, to make a more quantitative comparison between the experimental and the theoretical curves, we can identify for each curve (either theoretical or experimental) the wavelength of the local maxima, minima, and shoulders. This approach permits us to identify in the considered energy range the possible experiment–theory correspondences, aside from considering the exact strength of each structure. We identified 15 features in the experimental spectrum to be used for the comparison (column “Exp.” in Table 4).

By assessing the degree of overlap between the experimental structures and the theoretical ones, we find that the features of the computed absorption spectrum of Model 1-d show a good correspondence to 14 out of the 15 possible matches, while the absorption profile of PT shows a much lower (6 as compared to 14) number of possible correspondences with the experiments.

This point is of particular importance because it can support the hypothesis that the Model 1 tetramer of [47,48] could be one of the possible final aggregate forms of the DHI monomers initially present in the solvent within the optical experimental configuration previously described.

Of course, given the high degree of structural heterogeneity in eumelanin-type polymer and the lack of experimental evidence on the structure and redox state of main building blocks in DHI melanin, we must consider these data as being largely speculative. Further work is necessary to assess the effect of the solvent on the structural and optical properties of the model tetramers under investigation.

### 2.6. Adhesion Energy

**Figure 10:**
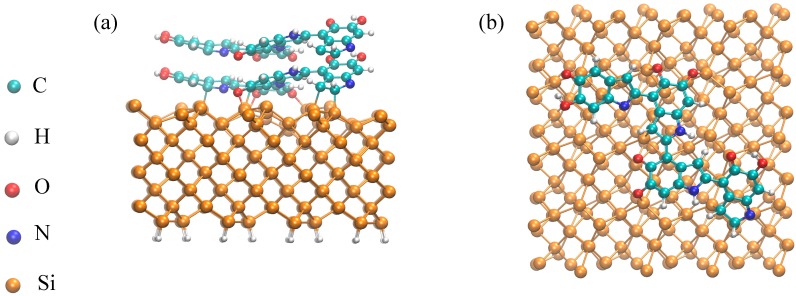
Adsorption of two stacked tetrameric models 1-d by Panzella [48] on a Si surface. The full system with the slab of the surface supercell used in the calculations is displayed: (**a**) side view; (**b**) top view. The bottom atoms are hydrogen-passivated.

Studies of the adhesion properties of eumelanin building blocks on the silicon surface were then carried out using Model 1-d. Figure 10 shows two views of adsorbed tetramer on silicon. The adhesion energies are calculated as the difference between the energy of the composite system and the sum of the energies of the components (non-interacting surface and molecule). The results are reported in Table 5.

Stronger adhesion properties are determined as the number of C=O groups in the molecules increases. Carbonyl (C=O) groups tend to bind to the surface, probably because of their expectedly greater polarization by resonance effects (the oxygen atom should be more negatively charged) and more efficient π–electron overlap (favored orbital interaction between surface and the π-type orbitals of the tetramer). If verified in future experiments, the computational finding that more oxidized forms of the oligomers exhibit greater adhesion properties can be of potential interest for improving the current coating/impregnation protocols for porous silicon. For example, the addition of oxygen gas or an enhancement of the oxidizing power inducing DHI polymerization over pSi may promote a faster and more efficient adhesion. This may be a valuable strategy to address current interface issues. It will also be of interest to assess whether interaction between oxidized DHI oligomers and the PSi surface can promote tautomerism between QM and IQ with an increase in the proportion of C=O groups at each degree of oxidation and further stabilization. These and other issues are the focus of ongoing work.

Moreover, from the comparison with the adhesion energies of stacked molecules (Table 6), it can be easily inferred that the process of stacking of multiple molecular structures upon the Si surface is energetically favorable, although less favorable with respect to single molecule docking. Hence, this result highlights the capability of eumelanin structural models to efficiently cover the inorganic surface, as expected in a hybrid interface for photovoltaic applications.

Future theoretical and experimental work aimed at completing these preliminary model experiments will hopefully improve our understanding of the eumelanin–silicon interface for possible applications in photovoltaics.

## 3. Materials and Methods

### 3.1. PSi–Eumelanin Hybrid Preparation

PSi structures were fabricated by electrochemical etching in the dark of *n*^+^ doped bulk Si wafers (Siltronix, Archamps, France) according to the procedure indicated in [11]. The pore filling was performed by impregnating the PSi pores with a DHI–EtOH solution, prepared as described in [12].

The electrochemical Impedance Spectroscopy measurements were performed using a PARSTAT 2273 potentiostat by Princeton Applied Research (Ametek, Berwyn, PA, USA). Each measurement was made using a constant current of 0.1 mA and a superimposed oscillating signal, whose amplitude was 10 µA, in the frequency range 100 kHz–100 mHz. The electrochemical solution was a 0.1 M KCl in EtOH. For the absorbance measurements, we prepared several DHI/EtOH solutions by adding DHI powder to EtOH in several concentrations: 0.5, 0.25, and 0.12 mg/mL. The measurements were performed at room temperature, using a quartz cell sealed with Parafilm^®^ and a PerkinElmer Lambda950 UV-Vis-NIR spectrophotometer (Perkin Elmer Italia Spa, Milano, Italy).

The electrochemical oxidation was performed using a 0.1 M KNO_3_ in EtOH solution according to the procedure described in [36]. The maximum oxidation is reached when, in a constant current configuration, the maximum applicable voltage of 10 V is reached. This corresponds, as mentioned in the results section, to an oxygen content equal to 50 at%, as measured by SEM-EDS.

The photocurrent measurements were performed using a halogen lamp as a white source and a Keithley (Keithley Instruments, Cleveland, OH, USA) 6487 multimeter to measure the current intensity. The illuminated spot radius on the sample was *r* = 2.5 mm. More details about the procedure can be found in [12].

### 3.2. Formation and Stacking Energies Calculations

Density–functional theory (DFT) calculations are performed using the plane–wave Quantum Espresso package [49].The Perdew–Burke–Ernzerhof (PBE) exchange–correlation functional [40,41] is used together with ultrasoft pseudopotentials [50] and Grimme correction to include dispersive forces in the system [51,52]. We used energy cutoffs of 20 and 250 Ry for the wave function and electronic density, respectively. Structures have been relaxed until all the atomic forces were smaller than 0.001 a.u. The calculations of the monomeric units and the complete tetrameric structures have been performed with a unit cell with the smallest linear dimension of 20 Å, thus leaving at least 8 Å of empty space between atoms in replicated cells. Periodic boundary conditions are applied to this large supercell, with the Brillouin zone sampled at the Γ point only.

In order to describe the effect of the solvent on the formation energy, a continuum solvation model was used: the self-consistent continuum solvation model (SCCS) proposed by Andreussi, Dabo, and Marzari [53] and implemented in the Quantum espresso Environ tool [49] has been adopted, giving an implicit description of the solvents (methanol and water in our case). The Quantum Espresso, opEn-Source Package for Research in Electronic Structure, Simulation, and Optimization), version used here is 6.1. The maintenance and further development of the Quantum ESPRESSO distribution is promoted by the Quantum ESPRESSO Foundation under the coordination of Paolo Giannozzi (Univ. Udine, Italy) with the strong support of the CINECA National Supercomputing Center in Bologna under the responsibility of Carlo Cavazzoni. Calculations of the adhesion energies of eumelanin protomolecules on the substrate were performed using a (100) slab of silicon atoms consisting of a 7 × 7 replica of the surface unit cell. In this case we sampled the Brillouin zone with a grid of 4 × 4 points within the Monkhorst–Pack scheme [54]. Different starting molecule–surface configurations have been considered. They have been used as input in geometry optimization procedures that have been performed by fully relaxing the positions of all of the atoms in a supercell, except for the atoms of the bottom layer of the semiconductor slab, which, instead, have been kept fixed to mimic the underlying bulk. The results that we will present refer to the minimum energy configurations.

### 3.3. Optical Spectra Calculation

DFT [55,56] and TD-DFT [57] computations were performed using the NWChem (PNNL Pacific Northwest National Laboratory (Richland, Washington state. USA), version 6.6) simulation package [58,59]. Geometry optimizations have been obtained using the Becke three-parameter Lee–Yang–Parr (B3LYP) hybrid exchange–correlation (XC) functional [60,61], in combination with the 6–31 G* basis-set, a valence double-ζ set augmented with d polarization functions.

The B3LYP exchange-correlation (XC) functional has stable behavior, with some well-documented limitations [62]. The structural optimization of each tetramer was performed without imposing symmetry constraints. 

To obtain the singlet–singlet excitation energies and the electronic absorption spectrum in the visible/UV region for each tetramer, TD-DFT calculations were performed at the same level B3LYP/6-31G* employed for its electronic ground-state. The frequency–space implementation of the TD-DFT scheme based on the linear response of the density matrix was used. In this computational approach, the poles of the linear response function are associated with vertical excitation energies and the pole strengths with the corresponding oscillator strengths [63].

For each molecule only the first 120 singlet–singlet roots, which in all cases were sufficient to cover the energy window considered here, were taken into account.

A similar computational scheme has been successfully applied on several other families of organic molecules and to another class of molecules that are the basic constituents of eumelanin [46,64,65,66,67].

## 4. Conclusions

By combining model experiments with different theoretical approaches, it has been possible to demonstrate that the eumelanin/PSi interface is partially stabilized by mild oxidation of the inorganic surface, probably generating an expanded range of functional groups amenable to efficient interaction with growing eumelanin building blocks. Model computational studies indicated, moreover, that the highest redox states of oligomer components in eumelanins are more susceptible to adhesion than the reduced forms. These results provide an improved background for the development of advanced eumelanin–PSi interfaces with better photocurrent stability properties for photovoltaic applications.

## Figures and Tables

**Table 1:** Formation energies [eV] of tetrameric model 1 in the four electronic configurations of Figure 4; values in a vacuum, methanol, and water are given.

Formation Energy (eV)	Vacuum	Methanol	Water
Model 1-a (one C=O)	−0.56	−0.12	0.67
Model 1-b (two C=O)	−0.26	0.35	0.74
Model 1-c (four C=O)	−1.20	−0.07	0.92
Model 1-d (six C=O)	−1.46	−0.20	0.91

**Table 2:** Stacking energies [eV] of tetrameric model 1 in the various oxidation states and tautomeric forms of Figure 8; values in a vacuum, methanol and water are given.

Stacking Energy (eV)	Vacuum	Methanol	Water
Model 1-a (one =O)	−1.52	−1.08	−0.89
Model 1-b (two =O)	−1.22	−1.01	−0.78
Model 1-c (four =O)	−1.75	−1.22	−0.88
Model 1-d (six =O)	−2.01	−1.76	−1.21

**Table 3:** Principal features of the calculated spectra for the PT and Model 1-d. For each structure we report (from top to bottom) the position of the onset peak (row C), the wavelength and energy of the first structure within the energy range considered (row B), and the position of the principal peak (row A). For each of these features, the electronic transitions that contribute to each structure are reported in the last column.

PT	C	Onset	*1086 nm (1.14 eV)*	HOMO → LUMO
	B	1st Peak in Range	*800 nm (1.55 eV)*	HOMO − 2 → LUMO
				HOMO − 2 → LUMO + 1
	A	Maximum Peak	*410 nm (3.02 eV)*	HOMO − 6 → LUMO + 2
				HOMO − 6 → LUMO + 1
				HOMO − 3 → LUMO + 1
				HOMO − 2 → LUMO + 3
Model 1-d	C	Onset	*1563 nm (0.79 eV)*	HOMO → LUMO
	B	1st Peak in Range	*690 nm (1.80 eV)*	HOMO − 1 → LUMO + 1
	A	Maximum Peak	*367 nm (3.38 eV)*	HOMO → LUMO + 4

**Table 4:** Positions in wavelength (nm) of the main structures of the absorption curves considered in the present work (i.e., the computed ones for the PT and Model 1-d structures and the experimental one). LM stands for a local maximum, Lm stands for a local minimum, and S stands for a shoulder. Data are listed from the low to the high wavelength values of the range considered here. In the column names, “N.” indicates the ordinal of the spectral structures, “Exp.” the experimental data, “Model 1-d” and “PT” the computed absorption structures for the Model 1-d and PT tetramers, respectively. [A] and [B] in the table refer to two absorption maxima defined in the text.

N.	Exp. (nm)	Model 1-d (nm)	PT (nm)	N.	Exp. (nm)	Model 1-d (nm)	PT (nm)
1	210 (LM)	217 (LM)	213 (LM)	--	--	--	384 (S)
2	220 (LM)	226 (LM)	220 (S)	10	400 (Lm)	392 (Lm)	--
--	--	--	226 (Lm)	--	--	--	410 (LM) [A]
3	230 (LM)	238 (LM)	240 (LM)	11	428 (S)	415 (S)	--
4	250 (Lm)	249 (LM)	259 (LM)	--	--	--	453 (Lm)
5	277 (S)	263 (S) 275 (S)	270 (LM)	12	456 (LM)	445 (LM)	--
6	298 (LM)	281 (LM)	292 (S)	--	--	--	485 (LM)
--	--	292 (Lm)	305 (Lm)	13	500 (S)	511 (LM)	--
7	305 (LM)	317 (LM)	319 (LM)	--	--	--	550 (S)
8	322 (S)	330 (S)	325 (S)	14	612 (S)	575 (S)	617 (LM)
9	340 (S)	340 (S)	340 (S)	15	700 (S)	690 (LM) [B]	700 (S)
--	--	367 (LM) [A]	355 (Lm)	--	--	--	800 (LM) [B]

**Table 5:** Adhesion energies (eV) of tetrameric Model 1 in the four electronic configurations of Figure 5.

Molecule	Adhesion Energy (eV)
Model 1-a	−5.37
Model 1-b	−6.73
Model 1-c	−7.85
Model 1-d	−10.99

**Table 6:** Adhesion energies (eV) of model tetramer structures (single and stacked molecules).

Molecule	Adhesion Energy (eV)—Single Molecule	Stacked Molecules
Model 1-a	−8.76	−8.23
Model 1-b	−8.93	−7.95
Model 1-c	−9.89	−9.02
Model 1-d	−11.02	−10.90

## Supplementary Materials

Supplementary materials can be found at www.mdpi.com/1422-0067/18/7/1567/s1.

## Abbreviations

PSi	Porous silicon
DHI	5,6-Dihydroxyindole
DHICA	5,6-Dihydroxyindole-2-carboxylic acid
DFT	Density Functional Theory
TD-DFT	Time-dependent DFT
EtOH	Ethanol
AISSP	Ammonia-induced solid state polymerization
SEM-EDS	Scanning Electron Microscopy—Energy Dispersive Spectroscopy
