# Supplementary material for: Deciphering Molecular Mechanisms of Interface Buildup and Stability in Porous Si/Eumelanin Hybrids
Source: Int J Mol Sci. 2017 Jul 19;18(7):1567. doi: 10.3390/ijms18071567 (PMC5536055; doi:10.3390/ijms18071567)
Supplement: Supplementary file 1 [file ijms-18-01567-s001.pdf]

Supplementary information

Deciphering molecular mechanisms of interface buildup and stability in porous Si/eumelanin hybrids

Elisa Pinna [1,2*], Claudio Melis [1,2,*] , Aleandro Antidormi [1,2] , Roberto Cardia [1] , Elisa Sechi [1] , Giancarlo Cappellini [1] , Marco d'Ischia [3], Luciano Colombo [1,2] and Guido Mula [1,2]

[1] Dipartimento di Fisica, Università degli Studi di Cagliari, S.P. 8 km 0.700, 09042 Monserrato (Ca), Italy – e-mail: guido.mula@unica.it

[2] Istituto Officina dei Materiali CNR-IOM, unità di Cagliari SLACS, Cittadella Universitaria di Monserrato, S.P. 8 km 0.700, 09042 Monserrato (Ca) – e-mail: luciano.colombo@dsf.unica.it

[3] Department of Organic Chemistry and Biochemistry, University of Naples "Federico II", Via Cintia 4, 80126 Naples, Italy - e-mail: dischia@unina.it

* Correspondence: elisa.pinna@dsf.unica.it - +39 070 6754787; claudio.melis@dsf.unica.it - +39 070 6754929

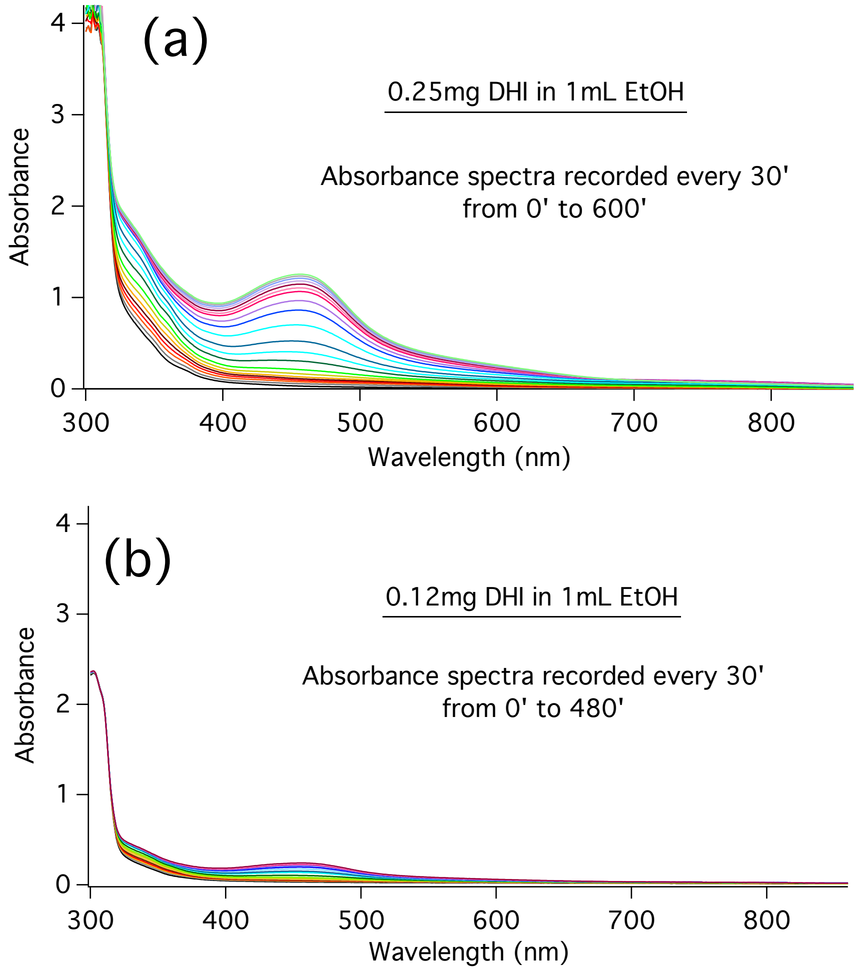

Figure S1: Absorbance spectra as a function of time for two different concentrations of DHI in EtOH. (a) 0.25 mg/mL and (b) 0.12 mg/mL. The vertical axis has been kept identical to that of Figure 5 for easier comparison.

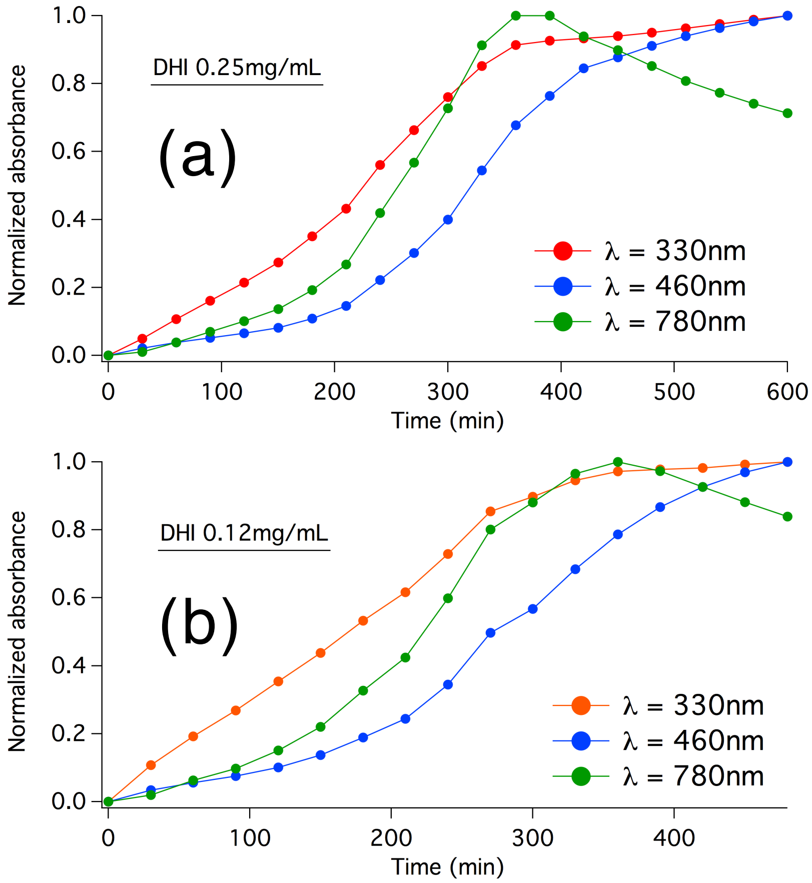

Figure S2. Analysis of the evolution of the absorbance spectra of the DHI in EtOH solutions shown in Figure S1 at three different spectral positions: 330 nm, 460 nm and 780 nm. The curves represent the normalized evolution of the absorbance for (a) 0.25 mg/mL and (b) 0.12 mg/mL.

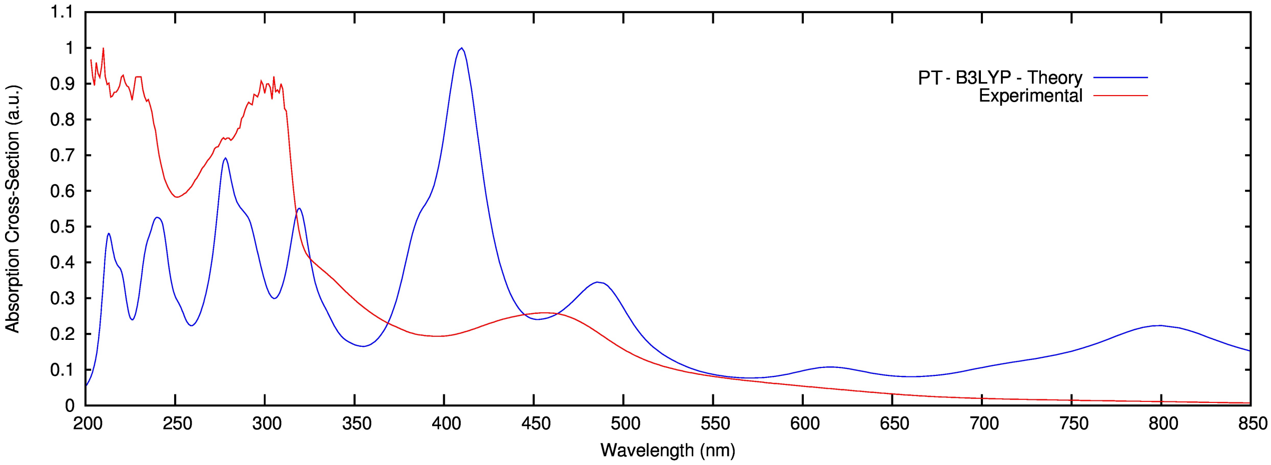

Figure S3. Comparison between the calculated spectra for the PT structure (blue line) and the experimental absorption of 0.25mg/mL solution of DHI in EtOH (red line, curve corresponding to the absorbance of after 600').

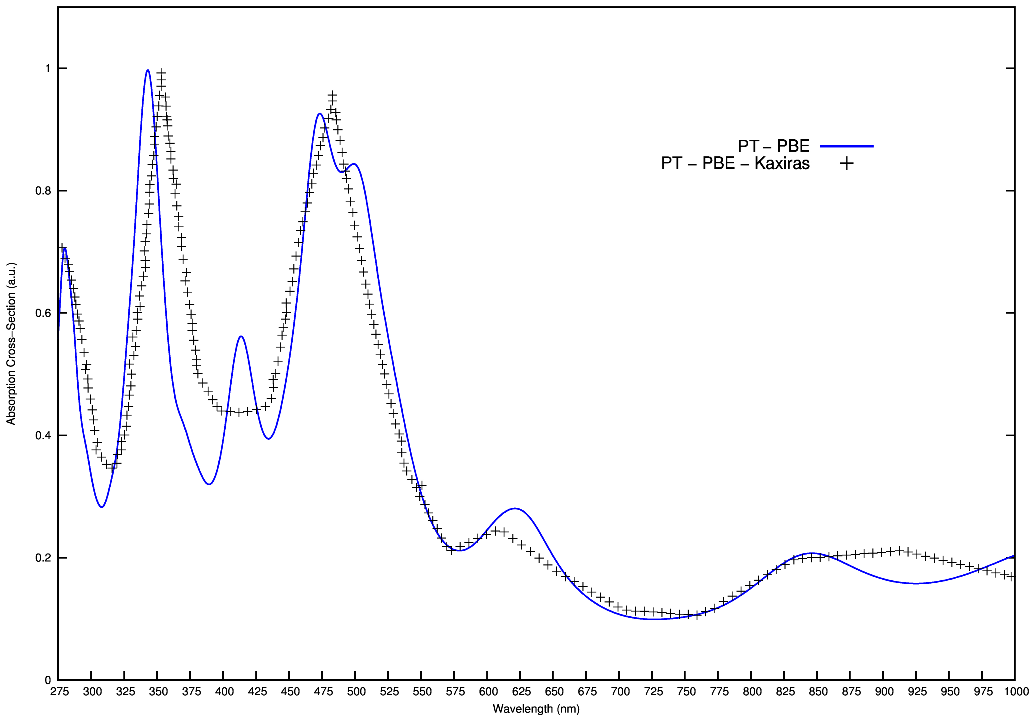

Figure S4. Comparison of the theoretical/computational spectrum for the PT structure calculated in this work with that reported by Meng and Kaxiras [44].
